# Editorial: Origin and Evolution of Hepatitis Viruses

**DOI:** 10.3389/fmicb.2021.740255

**Published:** 2021-08-25

**Authors:** Carla Osiowy, Lilly Yuen

**Affiliations:** ^1^National Microbiology Laboratory, Public Health Agency of Canada, Winnipeg, MB, Canada; ^2^Victorian Infectious Diseases Reference Laboratory, Melbourne Health, Peter Doherty Institute, Melbourne, VIC, Australia

**Keywords:** phylogeny, geographic distribution, recombination, host shift, resistance, genotype, subgenotype

Viral infection with hepatitis A, B, C, D, or E viruses (HAV, HBV, HCV, HDV, HEV) results in the syndrome of hepatitis, characterized by inflammation of the liver. Each virus is classified within a different virus family, yet all have hepatocyte-specific tropism and similar clinical manifestations. Full permissive infection with human hepatitis viruses is limited to higher primates, except for HEV, yet hepatitis-like viruses are known to infect invertebrates and all manner of vertebrates, including animals of the Laurasiatheria clade, such as bats and other insectivorous small mammals, suggesting an ancient origin and complex evolutionary history for hepatitis viruses. The definitive origin of these viruses remains largely unknown.

This Special Research Topic includes papers on HBV, HCV, HDV, and HEV, and includes studies investigating the consequences of virus evolution, such as geographic distribution and clinical outcomes. Other papers investigate intra-patient evolution, including super-infection and recombination, but also evolution over extensive timescales, thus providing a glimpse into hepatitis virus origins.

Several papers within the Topic presented important perspectives on the origins of HBV and HDV. The paper by Locarnini et al. elucidates the origin of primate HBV in the context of host evolution and migration. The authors posit that the evolutionary impetus giving rise to contemporary human and Old World non-human primate (NHP) HBV involved early human migration out of Africa during the upper Paleolithic era and the Neolithic agricultural expansion. The authors conclude that HBV evolution has occurred over many thousands of years with lineages disappearing over time and extant genotypes arising from specific population movements, such as slave trading. Most importantly, their investigation suggests that co-evolution among human and NHP HBV is not supported. The study by Netter et al. also investigated possible co-evolution of HDV and HDV-like agents with their hosts. In this important study, the authors advance the idea that HDV, a satellite virus most similar to plant viroids, likely originated within a cellular transcriptome as a circular RNA with ribozyme activity. Although delta-like agents have been detected in a multitude of different animals, including birds, fish, and insects, the helper virus, if indeed one is required, is not known. The paper suggests that host shifting, not co-divergence, is the suggested mode of HDV evolution based on HDV/host phylogeny.

Viral genomic recombination, as a mechanism of evolution involving viral superinfection, was the focus of several papers within the Research Topic. Jose-Abrego et al. observed that a high percentage (56%) of HIV-HBV co-infected patients were infected with multiple heterologous HBV genotypes (gt), including gtH, providing opportunity for genomic recombination. The paper from Giersch et al. presents an elegant study of HDV superinfection amongst gts1 and 3 using a human chimeric liver mouse model, observing that recombination is not a significant evolutionary process for HDV. Productive infection with multiple HDV strains, regardless of genotype, was not observed within a single cell, thus reducing the likelihood of recombination. Strain specific L-HDAg and/or the innate immune response induced during primary infection may play a role in suppressing superinfection. RNA recombination is thought to occur via the host polymerase switching viral genome templates; however, it is rarely observed in negative sense RNA viruses (Patiño-Galindo et al., 2021).

The distinct global distribution of hepatitis viruses provides evidence of ongoing evolution, with phylogenetic analyses delineating the relationship among viral genotypes. In the paper by de Bernardi Schneider et al. a novel quasi-subgenotype of HBV gtD was observed to be common among HBV-infected Inuit patients living in West Greenland communities. By use of Bayesian inference, the quasi-subgenotype was dated to approximately 629 CE, thus describing yet another unique HBV subtype identified within Indigenous populations (Bouckaert et al., 2017; Yuen et al., 2019), further confirming the sustained HBV/human host relationship. Designating new HBV subgenotypes requires that a specific genetic divergence criteria (≤4%) be met. The paper by Nicot et al. describes the basis for a similar criteria to differentiate HEV gt3 subgenotypes. By analyzing full genome (FG) HEV sequences, the authors generated a robust cut-off (0.093 nucleotide substitutions/site) to define a gt3 subgenotype. Following on the topic of HEV genotypes, the study by O'Keefe et al. describes the molecular epidemiology of HEV in Australia. The incidence of HEV within resource-rich regions has historically been associated with travel to endemic regions; however, gts3 and 4, associated with zoonotic transmission primarily from the animal family Suidae (hogs, boar, pig), now circulate within Australia, and thus are considered autochthonous. Following phylogenetic analysis, O'Keefe et al. determined that several gt3 non-travel associated strains may represent new “Australian” subgenotypes, and were likely transmitted from consumption of pork products.

Differences among hepatitis virus genotypes regarding pathogenesis, clinical, and treatment outcomes, have been described for HBV (Pujol et al., 2020), HCV (Sarrazin, 2021), and HDV, resulting in part from viral evolution and genomic mutation. The paper by Micas et al. describes an example of this with HEV, such that infection with gt4 was associated with higher ALT and AST activity than gt3 infection. This difference may be related to genotype-specific host innate and inflammatory responses. Both Araujo et al. and Rahimi et al. analyzed clinically significant viral hepatitis mutations. By parsing out clinically relevant immune escape, antiviral resistance and hepatocellular carcinoma-related mutations in >6,000 FG HBV sequences, Araujo et al. observed associations between specific mutation profiles and certain genotypes or subgenotypes. The study by Rahimi et al. investigated direct acting antiviral (DAA) resistance-associated substitutions (RAS) within the NS5A and NS5B genomic regions of HCV in treatment-naïve Iranian patients. They found different NS5A RAS prevalence among gts1a and 3a, although no NS5B RAS were observed, which is consistent with the reduced fitness of NS5B RAS in the absence of DAA.

Although the Special Research Topic did not include any studies of HAV, there have been important advancements in the past several years toward understanding the origin and evolution of HAV. Targeted exploration among non-primate mammals, such as bats, rodents, marsupials and harbor seals, has identified hepatovirus genus or HAV-like sequences, suggesting the capacity for HAV to infect diverse species (Anthony et al., 2015; Drexler et al., 2015; de Oliveira Carneiro et al., 2018; He et al., 2021). As observed with other hepatitis viruses, the HAV evolutionary pathway appears complex, involving host shifts (de Oliveira Carneiro et al., 2018) and a possible ancestral origin as an insect-borne virus (Drexler et al., 2015). This complex evolution may have resulted in a unique optimized host codon usage by the virus, resulting in increased capsid translational and protein-folding fitness in relation to other picornaviruses (D'Andrea et al., 2019).

The origin and evolution of hepatitis viruses continues to fascinate as evidenced by a continual increase in publications on the topic. As emerging and zoonotic viruses pose a serious threat to human health, a greater understanding of hepatitis-like viruses and human hepatitis virus evolution will help us prepare for future challenges. It is hoped that the Special Research Topic has contributed to this.

## Author Contributions

CO wrote the editorial. LY reviewed and provided feedback.

## Conflict of Interest

The authors declare that the research was conducted in the absence of any commercial or financial relationships that could be construed as a potential conflict of interest.

## Publisher's Note

All claims expressed in this article are solely those of the authors and do not necessarily represent those of their affiliated organizations, or those of the publisher, the editors and the reviewers. Any product that may be evaluated in this article, or claim that may be made by its manufacturer, is not guaranteed or endorsed by the publisher.

## References

[B1] Anthony S. J. St. Leger J. A. Liang E. Hicks A. L. Sanchez-Leon M. D. Jain K.. (2015). Discovery of a novel hepatovirus (phopivirus of seals) related to human hepatitis A virus. mBio 6:1180. 10.1128/mBio.01180-15 26307166 PMC4550696

[B2] Bouckaert R. Simons B. C. Krarup H. Friesen T. M. Osiowy C. (2017). Tracing hepatitis B virus (HBV) genotype B5 (formerly B6) evolutionary history in the circumpolar Arctic through phylogeographic modelling. PeerJ 5:e3757. 10.7717/peerj.3757 28875087 PMC5581946

[B3] D'Andrea L. Pérez-Rodríguez F. J. de Castellarnau M. Guix S. Ribes E. Quer J.. (2019). The critical role of codon composition on the translation efficiency robustness of the hepatitis A virus capsid. Genome Biol. Evol. 11, 2439–2456. 10.1093/gbe/evz146 31290967 PMC6735747

[B4] de Oliveira Carneiro I. Sander A. L. Silva N. Moreira-Soto A. Normann A. Flehmig B.. (2018). A novel marsupial hepatitis A virus corroborates complex evolutionary patterns shaping the genus hepatovirus. J. Virol. 92, e00082–18. 10.1128/JVI.00082-18 29695421 PMC6002732

[B5] Drexler J. F. Corman V. M. Lukashev A. N. van den Brand J. M. Gmyl A. P. Bruenink S.. (2015). Evolutionary origins of hepatitis A virus in small mammals. Proc. Natl. Acad. Sci. U.S.A. 112, 15190–15195. 10.1073/pnas.1516992112 26575627 PMC4679062

[B6] He W. Gao Y. Wen Y. Ke X. Ou Z. Li Y.. (2021). Detection of virus-related sequences associated with potential etiologies of hepatitis in liver tissue samples from rats, mice, shrews, and bats. Front. Microbiol. 12:653873. 10.3389/fmicb.2021.653873 34177835 PMC8221242

[B7] Patiño-Galindo J. Á. Filip I. Rabadan R. (2021). Global patterns of recombination across human viruses. Mol. Biol. Evol. 38, 2520–2531. 10.1093/molbev/msab046 33585889 PMC8136497

[B8] Pujol F. Jaspe R. C. Loureiro C. L. Chemin I. (2020). Hepatitis B virus American genotypes: pathogenic variants? Clin. Res. Hepatol. Gastroenterol. 44, 825–835. 10.1016/j.clinre.2020.04.018 32553521

[B9] Sarrazin C. (2021). Treatment failure with DAA therapy: importance of resistance. J. Hepatol. 74, 1472–1482. 10.1016/j.jhep.2021.03.004 33716089

[B10] Yuen L. K. Littlejohn M. Duchêne S. Edwards R. Bukulatjpi S. Binks P.. (2019). Tracing ancient human migrations into Sahul using hepatitis B virus genomes. Mol. Biol. Evol. 36, 942–954. 10.1093/molbev/msz021 30856252

